# Supplementary material for: Direct translation of climbing fiber burst-mediated sensory coding into post-synaptic Purkinje cell dendritic calcium
Source: eLife. 2020 Sep 28;9:e61593. doi: 10.7554/eLife.61593 (PMC7581426; doi:10.7554/eLife.61593)
Supplement: Transparent reporting form [file elife-61593-transrepform.docx]

***eLife’s* transparent reporting form**

We encourage authors to provide detailed information *within their submission* to facilitate the interpretation and replication of experiments. Authors can upload supporting documentation to indicate the use of appropriate reporting guidelines for health-related research (see [EQUATOR Network](about:blank)), life science research (see the BioSharing Information Resource), or the [ARRIVE guidelines](about:blank) for reporting work involving animal research. Where applicable, authors should refer to any relevant reporting standards documents in this form.

If you have any questions, please consult our Journal Policies and/or contact us: [editorial@elifesciences.org](about:blank).

**Sample-size estimation**

- You should state whether an appropriate sample size was computed when the study was being designed
- You should state the statistical method of sample size computation and any required assumptions
- If no explicit power analysis was used, you should describe how you decided what sample (replicate) size (number) to use

Please outline where this information can be found within the submission (e.g., sections or figure legends), or explain why this information doesn’t apply to your submission:

The sample size estimation was not performed due to the nature of the experiments. But sample size was determined based on previous studies that employed similar methodological approach and samples. These include:

**For *in vivo*,**

Najafi, F., Giovannucci, A., Wang, S. S. & Medina, J. F. Sensory-driven enhancement of calcium signals in individual Purkinje cell dendrites of awake mice. *Cell Rep* **6**, 792-798, doi:10.1016/j.celrep.2014.02.001 (2014).

Najafi, F., Giovannucci, A., Wang, S. S. & Medina, J. F. Coding of stimulus strength via analog calcium signals in Purkinje cell dendrites of awake mice. *Elife* **3**, e03663, doi:10.7554/eLife.03663 (2014).

**For *in vitro*,**
Changhyeon Ryu, Dong Cheol Jang, Dayoon Jung, Yong Gyu Kim, Hyun Geun Shim, Hyun-Hee Ryu, Yong-Seok Lee, David J. Linden, Paul F. Worley and Sang Jeong Kim STIM1 Regulates Somatic Ca2+ Signals and Intrinsic Firing Properties of Cerebellar Purkinje Neurons. *J Neurosci* **37**, 8876-8894, doi:10.1523/JNEUROSCI.3973-16.2017 (2017).

**Replicates**

- You should report how often each experiment was performed
- You should include a definition of biological versus technical replication
- The data obtained should be provided and sufficient information should be provided to indicate the number of independent biological and/or technical replicates
- If you encountered any outliers, you should describe how these were handled
- Criteria for exclusion/inclusion of data should be clearly stated
- High-throughput sequence data should be uploaded before submission, with a private link for reviewers provided (these are available from both GEO and ArrayExpress)

Please outline where this information can be found within the submission (e.g., sections or figure legends), or explain why this information doesn’t apply to your submission:

Replicate information was provided in figure legends as the number of cells, sessions and animals. No outliers were encountered and all data were analyzed.

**Statistical reporting**

- Statistical analysis methods should be described and justified
- Raw data should be presented in figures whenever informative to do so (typically when N per group is less than 10)
- For each experiment, you should identify the statistical tests used, exact values of N, definitions of center, methods of multiple test correction, and dispersion and precision measures (e.g., mean, median, SD, SEM, confidence intervals; and, for the major substantive results, a measure of effect size (e.g., Pearson's r, Cohen's d)
- Report exact p-values wherever possible alongside the summary statistics and 95% confidence intervals. These should be reported for all key questions and not only when the p-value is less than 0.05.

Please outline where this information can be found within the submission (e.g., sections or figure legends), or explain why this information doesn’t apply to your submission:

Statistical methods used were clarified where ANOVA test and P values were mentioned in Figure legends. They were also explained in the Method section. Data were reported to be significant if P < 0.05, P < 0.01, and P < 0.001.

(For large datasets, or papers with a very large number of statistical tests, you may upload a single table file with tests, Ns, etc., with reference to sections in the manuscript.)

**Group allocation**

- Indicate how samples were allocated into experimental groups (in the case of clinical studies, please specify allocation to treatment method); if randomization was used, please also state if restricted randomization was applied
- Indicate if masking was used during group allocation, data collection and/or data analysis

Please outline where this information can be found within the submission (e.g., sections or figure legends), or explain why this information doesn’t apply to your submission:

Basically, cells were allocated into experimental groups if they show healthy morphology and normal calcium activity. Other specific criteria were described in the Method section.

**Additional data files (“source data”)**

- We encourage you to upload relevant additional data files, such as numerical data that are represented as a graph in a figure, or as a summary table
- Where provided, these should be in the most useful format, and they can be uploaded as “Source data” files linked to a main figure or table
- Include model definition files including the full list of parameters used
- Include code used for data analysis (e.g., R, MatLab)
- Avoid stating that data files are “available upon request”

Please indicate the figures or tables for which source data files have been provided:

Source data were provided main figures with graphs and statistical tests.
